# Virtually the same, but remotely different: health professionals, parents and children’s experiences of remote out-patient consultations

**DOI:** 10.1093/rheumatology/keaf106

**Published:** 2025-02-18

**Authors:** Holly Saron, Gavin Cleary, Anthony Marson, Jenny Ainsworth, Jennifer Downing, John Sandars, Laura Whitty, Shabnam Cheetham, Ian Sinha, Bernie Carter, Clare E Pain

**Affiliations:** Faculty of Health, Social Care and Medicine, Edge Hill University, Ormskirk, UK; Department of Women’s and Children’s Health, Institute of Life Course and Medical Sciences, University of Liverpool, Liverpool, UK; Department of Paediatric Rheumatology, Alder Hey Children’s NHS Foundation Trust, Liverpool, UK; Institute of Systems, Molecular and Integrative Biology, University of Liverpool, Liverpool, UK; The Walton Centre NHS Foundation Trust, Liverpool, UK; National Institute for Health and Social Care, Applied Research Collaborative, North West Coast, Liverpool, UK; Experimental Arthritis Treatment Centre for Children, University of Liverpool, Liverpool, UK; Institute of Systems, Molecular and Integrative Biology, University of Liverpool, Liverpool, UK; National Institute for Health and Social Care, Applied Research Collaborative, North West Coast, Liverpool, UK; Faculty of Health, Social Care and Medicine, Edge Hill University, Ormskirk, UK; Experimental Arthritis Treatment Centre for Children, University of Liverpool, Liverpool, UK; Department of Paediatric Rheumatology, Alder Hey Children’s NHS Foundation Trust, Liverpool, UK; The Walton Centre NHS Foundation Trust, Liverpool, UK; Department of Women’s and Children’s Health, Institute of Life Course and Medical Sciences, University of Liverpool, Liverpool, UK; Faculty of Health, Social Care and Medicine, Edge Hill University, Ormskirk, UK; Department of Women’s and Children’s Health, Institute of Life Course and Medical Sciences, University of Liverpool, Liverpool, UK; Department of Paediatric Rheumatology, Alder Hey Children’s NHS Foundation Trust, Liverpool, UK

**Keywords:** paediatric, adolescent, multidisciplinary team, telehealth, qualitative, remote, virtual, JIA, co-production

## Abstract

**Objective:**

To explore experiences, benefits and concerns associated with remote (telephone/video) consultations from the perspectives of children and young people with juvenile idiopathic arthritis (JIA), their parents and health professionals who were members of a multidisciplinary team in a paediatric rheumatology setting.

**Methods:**

Qualitative design (Interpretive Description) utilizing observation of remote (telephone/video) consultations and remote follow-up interviews with children and young people (7–18 years) with JIA, their parents and health professionals. The setting was a tertiary paediatric rheumatology clinic in a hospital in Northwest England. Two groups of experts-by-experience (children, young people, parents) provided high quality input into study design and dissemination materials. Data analysis used reflexive thematic analysis.

**Results:**

Thirty-seven participants were observed (11 video, five telephone consultations): health professionals (*n* = 8); mothers (*n* = 11); fathers (*n* = 3); children and young people (*n* = 15). Parents (*n* = 7), children and young people (*n* = 8) and health professionals (*n* = 7) were interviewed. The overarching theme was that remote consultations were ‘virtually the same but remotely different’ to face-to-face hospital-based consultations. Four sub-themes were identified: ‘It’s a catch-up rather than a check-up’; ‘A sense of familiarity but a shift in dynamics’; ‘Minimizing disruption and burden’; and ‘Being ‘seen’ but seen differently’.

**Conclusions:**

Overall, remote consultations were viewed positively, bringing benefits to children, young people and parents. There was a notable transition in responsibility towards children and young people and/or their parents for reporting and recognizing disease flare, compared with face-to-face consultations. Optimizing the experience of remote consultations though better preparation, information and education for children, young people, parents and health professionals is needed.

Rheumatology key messages:

- For children and young people with stable disease, remote consultations saved time, money and meant they missed less school.
- Remote consultations were perceived by children and young people and/or their parents as increasing their responsibility to be clear reporters/assessors of JIA.
- Our co-created resources may better prepare children, young people, parents and health professionals for successful remote consultations.

## Introduction

Government drivers within the UK have transformed telehealth services, offering digital alternatives to face-to-face consultations [1]. However, adopting digital technologies can disrupt how people operate and interact [2], requiring reconsideration of the consultation pathway in terms of resources, context and requirements [3]. Much of the recent guidance [4, 5] and research addressing remote paediatric consultations [6–8] was undertaken during the pandemic.

Across paediatric healthcare, advantages of remote (telephone/video) consultations include socio-economic benefits to families [9–12]. Other benefits include convenience [5, 8, 13], decreased patient stress [11], children feeling more comfortable at home [12] and a reduced healthcare carbon footprint [14]. Typically, if the context is right, parents [7] and children [15] express a preference for remote consultations, preferring video to telephone consultations [15, 16]. Wider benefits include reduced consultation/waiting times [12], improved clinic punctuality [8], reduced non-attendance rates [9, 10], lowered hospital utilization [10, 17] and increased patient throughput [18]. However, concerns exist regarding safeguarding [4], perpetuating digital inequality [5, 19], reduced ability to undertake physical examinations [8, 13], diminishment of the therapeutic relationship [12, 19, 20], more adult-centric practice and lowered child engagement [5, 12].

Juvenile idiopathic arthritis (JIA) is the most common rheumatological inflammatory disease of childhood [21], managed by a multidisciplinary team, with outpatient follow-up typically 3–6-monthly with ad hoc reviews for disease flares. Within paediatric rheumatology, remote consultation studies have typically used surveys [22–24] or randomized controlled trials [25]. However, robust experience-based studies from the perspectives of children, their parents or the multidisciplinary team are scarce.

This study aimed to address this deficit by exploring the experiences, perceived benefits and concerns of remote (telephone/video) consultation from the perspectives of children/young people with JIA, parents and health professionals who were members of an multidisciplinary team in a paediatric rheumatology setting.

## Methods

A qualitative Interpretive Description [26] study utilizing observation and interviews reported in accordance with COREQ guidelines (Supplementary Data S1, available at *Rheumatology* online) [27].

### Involvement of experts-by-experience

Two groups of experts-by-experience provided high quality engagement and gave us confidence not to pilot the methods (Supplementary Data S2, available at *Rheumatology* online).

### Recruitment and consent

A purposive sampling matrix (Supplementary Data S3, available at *Rheumatology* online) targeted children 7–18 years, based on diagnosis, type of consultation (telephone/video) and index of deprivation. Recruitment occurred following the major challenges associated with COVID-19 (7 January 2022 to 10 November 2022) when clinical practice was still undergoing challenges (e.g. cancelled clinics) (Supplementary Data S4, available at *Rheumatology* online, recruitment and stable in disease).

Interested parents agreed to share their contact details with the research team and were contacted by H.S. by phone/text, and then by email to share information sheets and schedule a call to discuss the study and obtain and document verbal consent/assent. Consent/assent were ongoing and checked at every interaction.

All data collection was undertaken by H.S. (for characteristics of H.S., Supplementary Data S1, available at *Rheumatology* online).

### Phase 1: Non-participant observations of telephone or video consultations

Non-participant observation [28] of video consultations was undertaken using the NHS video-calling platform, ‘Attend Anywhere’. Non-participant observation of telephone consultations occurred on-site at the hospital in a private room/office using a speaker phone; H.S. could observe the health professional but not the child/parents.

An observation sheet based on the Paediatric Consultation Assessment Tool [29] supported documenting field notes (Supplementary Data S5, available at *Rheumatology* online) on building the relationship, initiating the session, communication, gathering information, physical examination, planning, closure, issues relating to health inequalities, socio-technical aspects and anything specific to follow-up at interview. The researcher spoke only at the start (hello, re-confirm consent/assent), and at the end (thank you, reminder of follow-up interview). In video calls, the researcher switched off her camera and microphone after greeting the child and/or parent and re-confirming consent.

Demographic and disease-specific factors were recorded.

### Phase 2: Interviews with health professionals, parents, children and young people

Semi-structured interview guides were developed for children/young people (Supplementary Data S6, available at *Rheumatology* online), parents (Supplementary Data S7, available at *Rheumatology* online) and health professionals (Supplementary Data S8, available at *Rheumatology* online). Interview topics included information about themselves/their child, personal use of technology, and about the telephone/video consultation. Ideally both child and parent would agree to be interviewed, although one member of the dyad could decline. Where both agreed, parents and children were given the choice to be interviewed separately or together; they were only interviewed once. health professionals who were observed were interviewed.

Interviews were scheduled by email and/or text. Activity booklets (Supplementary Data S9, available at *Rheumatology* online) provided an outline of the questions to be asked and aimed to prepare the children. All interviews were audio-recorded using either a study-specific telephone or Microsoft Teams/Zoom. Beyond the obvious difference of being able to observe the child/parent in video interviews, both interview techniques appeared effective.

### Ethics and governance

Ethics approval was obtained from HRA and Health and Care Research Wales (HCRW) (IRAS ID 302805) as well as via the Health Research Ethics Committee, Edge Hill University. Security of consultations, storage of data, confidentiality and anonymity complied with UK GDPR [30], e.g. removing personally identifying details to avoid ‘deductive disclosure’ [31]. All participants were made aware of how we planned to protect anonymity via specific Participant Information Sheets (Supplementary Data S10, available at *Rheumatology* online).

### Data analysis

Handwritten observation/field notes were transcribed digitally. Interviews were transcribed verbatim by a secure approved professional transcription service (Supplementary Data S11, available at *Rheumatology* online), ensuring data quality. No qualitative analysis software was used. Preliminary data analysis was undertaken by H.S. using reflexive thematic analysis [32]. Preliminary themes were discussed within the research team and wider group of hospital-based health professionals to help ground findings in data and practice. Further cycles of thematic refinement occurred (H.S., B.C.).

Quotations are coded: D-Doctor, N-Nurse, O-Occupational Therapist, M-mother, F-father, B-boy, G-girl; digit indicates consultation [1–16] (e.g. B-8, boy, consultation 8).

## Results

### Demographics

Thirty-seven people participated: children and young people [*n* = 16; male (*n* = 7), female (*n* = 9), age range 7–18 years, median age 12 years] (Table 1), parents (*n* = 14) and health professionals (*n* = 8) (Table 2). No families who were contacted refused to participate; some did not respond to initial communication (reasons unknown).

**Table 1.** Patient characteristics (*n* = 16)

Patient characteristics	Number (%)
Male	7/16 (43.75%)
Female	9/16 (56.25%)
JIA Subtype	
Persistent oligoarthritis	4/16 (0.25%)
Extended oligoarthritis	1/16 (6.25%)
Polyarthritis (RF negative)	5/16 (31.25%)
Polyarthritis (RF positive)	2/16 (12.5%)
Systemic JIA^a^	2/16 (12.5%)
Enthesitis related arthritis	1/16 (6.25%)
Psoriatic arthritis	1/16 (6.25%)
Current treatment	
None	5/16 (31.25%)
DMARD	1/16 (6.25%)
Biologic	9/16 (56.25%)
DMARD and biologic	1/16 (6.25%)
Number of consults with HP previously	
First consult	3/16 (18.75%)
1–5	3/16 (18.75%)
>5	10/16 (62.5%)
Age (years)	Median 12
	Range 7–18
Ethnicity	
White British	10/16 (62.5%)
Other white background	1/16 (6.25%)
Indian	2/16 (12.5%)
Pakistani	1/16 (6.25%)
African	1/16 (6.25%)
Not recorded	1/16 (6.25%)
Index multiple deprivation^b^	
High	3/16 (18.75%)
Other	13/16 (81.25%)

a Diagnosis changed during study from systemic JIA to chronic nonbacterial osteitis. Fifteen participants were diagnosed with JIA over 12 months previously whilst one patient was diagnosed within the last 3 months (occupational therapist consultation).

b High indicates those in deprivation deciles 1–2 and other indicates those in deprivation deciles 3–10.

DMARD: disease modifying anti-rheumatic drug; HP: health professional; JIA: juvenile idiopathic arthritis; RF: rheumatoid factor.

**Table 2.** Overview of parent and health professional demographics and participant engagement within consultations

Parents (*n* = 14)	
Role	Mothers (*n* = 11)
	Fathers (*n* = 3)
Health professionals (*n* = 8)	
Role	Consultant doctor (*n* = 4)
	Resident (trainee) doctor (*n* = 1)
	Clinical nurse specialist (*n* = 2)
	Occupational therapist (*n* = 1)
Consultation (*n* = 16)	
Type of consultation	Video (*n* = 11)
	Telephone (*n* = 5)
Who attended consultation	Child/young person accompanied by parent (*n* = 13)
	Young person unaccompanied by parent (*n* = 2)
	Parent unaccompanied by child (*n* = 1)
Interviews	
Participation in interviews	Children and young people (*n* = 8); phone (*n* = 2), video (*n* = 6).
	Children and young people accompanied by parent (*n* = 7), unaccompanied by parent (*n* = 1)
	Parents (*n* = 7); mothers (*n* = 6), father (*n* = 1)
	Health professionals (*n* = 7). (Note: one doctor participated in three interviews, one nurse participated in two interviews, five health professionals participated in one interview.)
	Interviews lasted 20–40 min.

Sixteen consultations were observed (video *n* = 11, telephone *n* = 5); 2/5 telephone consultations were converted from video consultations due to poor video connectivity. In 6/11 video consultations, examination of joint range of movement was undertaken. Health professionals included consultant doctors (*n* = 4), resident (trainee) doctor (*n* = 1), clinical nurse specialists (*n* = 2) and an occupational therapist (*n* = 1); of these, seven were interviewed (10 interviews in total) (Table 2). Most parent participants were mothers (*n* = 11), three were fathers; of these, interviews were undertaken with mothers (*n* = 6) and a father (*n* = 1). Eight children/young people participated in interviews via telephone (*n* = 2) or by video (*n* = 6). Parents of two young people (17 years, 18 years) agreed for their child to be interviewed but were not interviewed themselves (Table 2). Interviews lasted 20–40 min.

### Overview of findings

**Figure 1.**
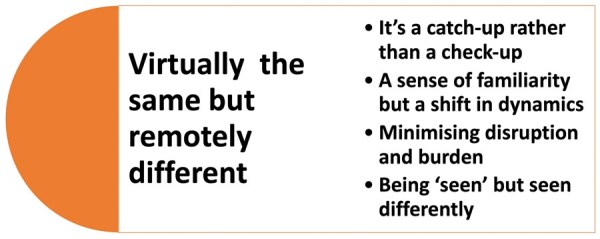
Overview of themes

The overarching theme was that remote consultations were ‘virtually the same but remotely different’ to face-to-face hospital-based consultations. Four sub-themes were identified (Fig. 1, Table 3); no diverse cases were identified.

**Table 3.** Additional quotations linked to themes

A catch-up rather than a check-up	Children, young people and parents I know it’s just been a fact of catching up, making sure I’m all right with everything (B-8). When she was in remission we could just check in and see how we’re getting on (M-9). I wouldn’t be able to get there [alone] (G-14). Health professionals [These stable children are typical of] more than half of the patients with JIA in follow up (D-13). More of a catch-up than a check-up (D-15). I have some patients that say shall we catch up every three months by video but then I’d still like to see you face to face once a year or something. Just so that they’re clear and I’m clear that actually for three months after consultation and then we’ll see you for a proper consultation once a year (D-12). If I’m worried about anything I can follow up (N-9). I think it went well and I’m confident that I made the same plan with them as what I would have done if they were in front of me (N-5). Some parents arrange for us to talk to the kid at school, they’ll go ‘I’ll have a quick word first then you can ring him on this number, he’s waiting for you’ (N-10).
A sense of familiarity but a shift in dynamics	Children, young people and parents [It] wasn’t as bad [as hospital-based consultations] … [but harder than chatting to friends who] I know much better (B-12). They’re much better [than hospital-based consultations] (G-14). It was thorough, just like every appointment when we’ve been seen at the hospital (M-8). When you’re face to face it’s more about [child], but when it’s on the phone it’s just me telling them about them (M-9). The pressure is on us [as parents] to monitor and notice things (M-9). [I’m] confident that the [remote] consultation covered everything it needed to (M-16). Health professionals There’s not much difference, I probably say and do most of what I say and do when I’m not on the phone (when I’m in clinic) (D-15). [I prefer] telephone appointments [as simpler technology] … [although] it’s much nicer to be able to talk with a family when you can see them (D-1). For some patients it’s fine and they’re very sensible and they would know when there’s an issue and they would contact us accordingly (D-12).
Minimizing disruption and burden	Children, young people and parents We [child, mother and father] used to go together, now it’s just mostly me and mum when we’re at home (B-8). [It’s] also the money, not that money is a huge issue for us. But we worked it out that we were probably spending £20–£25 a trip (M-2). [Our] three-hour round trip [becomes a telephone appointment lasting] 15 min … or even less (M-1). [My child doesn’t need to] miss another day of school or spend the day in a hospital (M-16). [Attending face-to-face consultations] It is having an impact on her schooling … to have her back for the afternoon, I just think was just brilliant (M-2). Health professionals They can’t necessarily afford to get to the hospital, which is an issue in itself to be seen face to face. If we don’t need them to travel in, why should they? (N-5). I think now, technology is so huge that even the poorest of families tend to have access, whether that’s through support from school, or different programmes (O-2).
Being ‘seen’ but seen differently	Children, young people and parents [A] lot less stuff [was checked] than the face-to-face (B-8). I mean, the only other thing was she had a look at my handwriting when I was there, whereas that wasn’t done this time (video consultation). But she said I could go in [to hospital], if I needed to have that done (G-2). at least being seen in person, you can have that reassurance that they’ve seen you … sometimes with a video call or voice call, it’s not as practical (G-14). there wasn’t nothing major that she would have got any more benefit from being there face-to-face (M-16). Health professionals the description … that whoever it is you’re talking to gives you … but it’s not enough sometimes (N-10). We have to see these children and see and feel their joints (N-5). [Rheumatology is] an art and science, it’s not statistics, it’s not ‘your blood sugar is this’, it’s hands-on (N-5). you can’t just speak to somebody, you need to see a joint and move the joint to see what’s happening to it (N-5).

### It’s a catch-up rather than a check-up

Everyone expected the consultations to be routine as the children were ‘stable in their disease’ (D-13) and perceived by health professionals to be the right population for remote consultations providing they are ‘able to access and use the technology that’s required’ (D-11) and if ‘they’ve got a stable disease and stable treatment, it’s perfect to have that contact via video’ (D-3). It was clear that routine remote consultations ‘can be one way, but … not the only way’ (N-10), were ‘a good option for families’ although there should be a ‘proper consultation [face-to-face] once a year’ (D-12).

Perceptions that consultations were routine may have framed the sense of them being more of a ‘catch-up and a chat about how it’s all going’ (M-13) suggesting that ‘if it’s over the phone, we’re not particularly worried about them’ (N-10).

Perceiving remote consultations as less significant than face-to-face consultations changed the practice of attendance, creating opportunities for parents and children/young people to break traditional ‘rules’ of attendance. In one telephone consultation the child was absent, and their parent attended the consultation without them; health professionals reported children opting out as ‘common’ thus leaving parents to ‘deal with’ (N-10) consultation.

The ability to exploit remote consultations meant children/young people and parents did not need to be in the same space to engage; children/young people could engage from school and their parents from work/home. With no need to be driven to the hospital, young people could attend routine appointments on their own.

The routine nature of the remote consultation meant that health professionals reported confidence in achieving the desired outcomes at a level congruent with a hospital-based consultation. Typically, the outcome was a routine follow-up appointment. No child required an urgent hospital-based follow-up consultation.

### A sense of familiarity but a shift in dynamics

Typically, children/young people, parents and health professionals reported being comfortable having/conducting telephone/video consultations as the format was like previous face-to-face experiences. Familiarity arose from the consistent, predictable clinical structure that helped parents feel confident. Mostly children talked of being comfortable as they ‘knew what would happen’ (G-14), boosting their confidence that ‘nothing was missed’ (G-16), and proposing remote consultations were the way forward, preferring ‘video appointments in the future and no face-to-face appointments’ (G-16).

However, remote working seemed to shift the dynamics of the consultation, placing the balance of responsibility onto children and parents to ensure thoroughness:

*‘I think they should see us more in clinic and less on video … what if I miss something, it sort of makes me do their job, and I’m not qualified’ (G-14).*

Parents worried about a sense of increased responsibility:

*‘On the phone I just worry I’m not making it sound as important as it is … like what if I don’t tell them something’ (M-13).*

Health professionals acknowledged the important role parents played in remote consultations and the trust they place in parents being able to recognize signs and symptoms of change as they ‘know the difference between a “flare” and a joint that is just “aching”’ (N-10).

Despite the remote consultations unfolding in an expected manner, they relied to a greater or lesser degree on familiarity, confidence with the technology (e.g. devices), software platform (most had experience of Zoom but not Attend Anywhere) and experience of remote engagement in everyday life such as having non-clinical video chats. However, even for children who were digitally confident, engaging remotely with a health professional was more challenging than chatting to friends. For some children this created a sense of difference from routine face-to-face consultations right from the start of their consultation.

Health professionals with limited ‘time to set up new tools’ (D-1) or who felt constrained by their perceived ‘lack of IT skills’ (N-9) preferred telephone consultations to video consultations.

### Minimizing disruption and burden

Prior to remote consultations, travel to the hospital was a routine, albeit inconvenient ‘part of life with a child with JIA’ (M-16). However, all participants acknowledged the key benefits accruing from telephone/video consultations.

In comparison to hospital-based consultations, telephone/video consultations were quick and required little additional time input from parents or children/young people and minimized disruption. Parents talked of trips to hospital taking ‘half the day out of you’ (M-13) and requiring ‘taking the day off work or adjust[ing] my work’ (F-8). Conversely, video calls were easier, and generated benefits as they did not require ‘[taking] time off and travelling’ (M-16) or ‘missing school’ (G-16).

Reducing the impact on school time was important for parents. One supportive school helped minimize disruption as ‘they let us sit in a little room’ (M-9) to have the remote consultation. Children/young people were equally enthusiastic about reducing impact on school time, saying remote consultations mean ‘I miss less school’ (G-2).

Hospital-based consultations included diverse travel and other costs such as ‘it’s not just the cost of the car park, it’s the petrol, they’ve all gone up!’ (M-2) and:

*‘we’ll have a Costa, then the appointment, then I’ll need to feed him because he’ll have missed lunch at school … it’s all extra’ (F-8).*

Health professionals reported being aware of the cost and time savings associated with remote consultations.

However, remote consultations changed some experiences that the children enjoyed. Hospital attendances could be a family affair with both parents attending or opportunities to spend time together on the journey ‘we used to have McDonald’s’ (B-8) and ‘he’d tell me what he thought about what [Doctor’s name] had said’ (F-8).

### Being ‘seen’ but seen differently

The routine of going to see, be seen and undergoing a hands-on physical examination was shifted in remote consultations. Consultation dynamics shifted as none of the children underwent hands-on examination and some (telephone consultations) were not actually seen, meaning visual physical cues were absent and health professionals relied on parental or child descriptions although ‘it’s not enough sometimes’ (N-10). One mother said telephone consultations were:

*‘pointless … I feel like you can say anything on the phone … It’s not the same as being seen because when … we have an actual appointment, he’ll look at him and check his joints and stuff’ (M-9).*

A limited repertoire of physical examination occurred in most video consultations, despite health professionals not having a direct hands-on approach, such as doing ‘activities with my hand’ (G-2) or ‘movements and stretches’ (G-13). The more limited repertoire was viewed positively by some children who were ‘happy not to have to do too many physical things’ (G-14) as these ‘make [my joints] hurt’ (G-16). However, some children expressed reservations about the limits of these hands-off physical examinations suggesting that they were not as complete or ‘thorough’ (B-8) and ‘were quite abrupt and quite short … about a minute or two’ (B-12) whereas hospital-based examinations were a ‘full body check-up’ (B-12). Although parents accepted the limitations of remote consultations, one mother worried because:

*‘[parents] don’t notice every flareup. There’s been occasions when we’ve gone in [hospital], she’s having a flareup in one particular joint, but then when she’s been reviewed, she’s got other joints flaring that we weren’t aware of’ (M-13).*

No health professionals expressed concern about not being hands-on in these remote consultations, although they noted that telephone consultations were more limiting and reported that in some situations ‘it’s much more difficult to feel like you’ve done a proper examination’ (D-12).

## Discussion

This was the first in-depth qualitative study to explore experiences, benefits and concerns associated with remote (telephone/video) consultations from the perspectives of children/young people with JIA, their parents and health professionals. In summary, our findings show that remote consultations brought benefits to children/young people and parents although some limitations were identified compared with face-to-face consultations. We co-created free-to-download resources (not yet evaluated) consisting of an animation (Remotely Ready: How To Get The Best Out Of Virtual Appointments) and information sheets targeted at children/young people, parents and health professionals that provide clear advice about preparing for telephone/video consultations.

Our findings show that for children/young people who were ‘stable in disease and treatment’, remote consultations brought benefits and were mostly welcomed across the participant groups. The importance of patient selection for remote consultations is widely echoed in the literature which reiterates the need for the aim of consultations to be relatively simple [33] and with appropriate patients (e.g. those with less complex needs) [34].

Our findings reflect many benefits previously reported as results of surveys and objective measures, such as reduced travel time and financial burden [23, 24, 33], enhancing school attendance [33], reducing parents’ time away from work and time efficiency for health professionals [33]. As with other studies, the preference across all groups was typically for video rather than telephone appointments [35]. Our findings confirmed that voice-only (no non-verbal cue) consultations limited health professionals’ ability to visualize the child’s body; arguably a crucial component of clinical assessment for some specialities or consultations [7, 35]. However, despite these constraints our health professionals felt confident in undertaking the consultations, perhaps reflecting their expertise and experience in paediatric rheumatology. However, this confidence in remote consultations is not a given; telephone consultations have been reported as being particularly challenging for medical students and trainees [36], although specific training can support learners’ confidence [37, 38] and is now being embedded in UK-based postgraduate paediatric training for doctors [39].

Even when children/young people can be seen, as in video consultations, issues arise. Children/young people, parents and health professionals all noted the challenges of the ‘corporeal conundrum’ [37] of consultations where physical in-contact examination cannot occur, noting that this creates a gap in what can be achieved. As with all disruptive technologies, remote consultations create shifts in usual working patterns and expectations for all parties. We noted the shifts in how clinical examination was undertaken and the dynamics of the consultation and acknowledge that core values (e.g. trust, self-efficacy), cornerstones to any consultation, are key for adoption of remote consultation by patients [2]. Sociocultural technical issues such as digital equity/inequity [40], digital inclusion [41] and access to/familiarity with the required technology [22] need consideration. The digital domain has been described as ‘a “super” social determinant of health’ (p657) [40]. Addressing service user challenges (e.g. limited access to technology, low internet speeds) [42, 43] is crucial, especially if digital health services are to prevent paradoxically exacerbating the health inequalities gap [44].

As previously reported [35], we noted that occasionally children did not attend consultations as parents had prioritized schooling over attendance. Although we did not note a diminishment in child engagement in the consultations when the child attended, other studies have raised concerns about reduction in the engagement of the child [45] and parent [34]. The risk to the clinical relationship and the quality and depth of communication in remote consultations has been identified [46]. Parents in our study and one young person perceived they had to ‘pick up’ health professionals’ work through a heightened responsibility to be clear, comprehensive reporters/assessors and not miss anything of importance; such concerns can underlie a preference for ‘real’ consultations. However, concerns may be mitigated by careful preparation of all stakeholders, using resources such as our information sheets. Concerns about the completeness of remote consultations are echoed by health professionals in other studies who have raised the importance and need for safety netting in remote consultations [47]. Careful reflection is needed when considering the value and suitability of remote consultations within paediatric rheumatology as they have the potential to disrupt the triadic alliance that is core to effective therapeutic relationships [12, 19, 20], particularly with long-term patients who are typically identified as the ideal target group for remote consultation.

Confidence and expertise in remote consultations requires investment in improved clinical evaluation tools and training packages [48], prioritizing telehealth in curricula [37, 39] and investment in actions to mitigate risks inherent in remote consultations [46]. Concerns have been raised about the quality of learning for trainee health professionals in remote consultations [36–38, 49]; however, this was not raised by the one resident doctor in our study.

As consultations shift to being ‘webside’ [49] new techniques to assess physical health (e.g. height and weight) are needed; routine collection of patient-reported outcome and experience measures may require development of novel digital solutions.

Drivers for satisfaction in remote consultations and telehealth for children/young people and parents are not clearly defined and agreed [24]. However, core components such as the clinical relationship, and confidence about the completeness and safety of the consultation are paramount for children/young people, parents and health professionals. Some children may be particularly vulnerable as a result of more aggressive disease [21], safeguarding issues and potential health inequalities arising from deprivation; where present, such factors may preclude remote monitoring and consultations.

### Strengths and limitations

This is the first study, to our knowledge, that uses a robust qualitative methodology to explore the experiences, perceived benefits and concerns of three key stakeholder groups in remote paediatric rheumatology consultations. The robustness of the study is built on careful engagement with experts-by-experience who were crucial in informing key aspects of design and dissemination. Our engagement with children/young people recognized their agency and we were highly reflexive in our approach (Supplementary Data S12, available at *Rheumatology* online, reflexivity).

However, this study is limited by a relatively small sample size, being specific to and being conducted in one NHS trust. Our dataset was too small to compare experiences in relation to other rheumatology or health conditions, or to other factors. Further, the fact that none of the children/young people required a face-to-face follow-up may reflect that selection had been too conservative. All patients who had been offered a remote consultation were screened. However, only certain patient groups were offered remote consultations as part of clinical care (e.g. those who had agreed to be scheduled, not ‘flaring’ and were able to access a remote consultation). This ‘pre-selection’ of patients requires consideration when interpreting the results of this study.

### Implications for practice and research

Recommendations include the need for better preparation, information and education (e.g. via our resources) for all involved in remote consultations to increase confidence, reduce challenges and support future digital directions. There are implications for partnering with education settings to provide appropriate spaces for remote consultations to minimize absence from education.

Future research priorities need to be defined by patients and parents. Such studies might include focusing on shared decision-making around choice of modality; the effectiveness of remote consultations in managing less ‘stable’ or more complex conditions/situations; assessing the longitudinal effectiveness of remote consultations; and supporting the quality of remote assessments (e.g. innovations such as novel digital patient-reported outcome measures, technology for remote examination of joints, and information gathered from wearable devices).

## Conclusion

Overall children/young people, parents and health professionals reported positive experiences of ‘being seen’ remotely. For children/young people whose condition was stable such as those in our study, they saved time, money and missed less school; limitations to physical examination were identified. The perception that children/young people and/or their parents felt a higher level of responsibility for reporting and recognizing disease flare, compared with face-to-face consultations is an area for further consideration.

## Supplementary material

Supplementary material is available at *Rheumatology* online.

## Supplementary Material

keaf106_Supplementary_Data

## Data Availability

Most data is available in the manuscript and supplementary data except for the full transcripts of the interviews; due to ethics approval constraints, access to the transcripts will be considered on a case-by-case basis.
