# Supplementary material for: Virtually the same, but remotely different: health professionals, parents and children’s experiences of remote out-patient consultations
Source: Rheumatology (Oxford). 2025 Feb 18;64(6):3361–9. doi: 10.1093/rheumatology/keaf106 (PMC12107072; doi:10.1093/rheumatology/keaf106)
Supplement: keaf106_Supplementary_Data [file keaf106_supplementary_data.pdf]

S1: COREQ (COnsolidated criteria for REporting Qualitative research) Checklist

Topic	Item no.	Guide Questions/'Description	Reported in section	Commentary
**Domain 1: Research team and reflexivity**				
**Personal characteristics**				
Interviewer/facilitator	1	Which author/s conducted the interview or focus group?		The first author (HS) conducted all observations and interviews.
Credentials	2	What were the researcher's credentials? E.g. PhD, MD	Phase 1: Non-participant observations of telephone or video consultations section p5<br>.	HS has a PhD and is a research fellow with an interest in children, young people and family experiences of health and healthcare. HS (non-clinical, white female, social scientist, PhD, working as Research Fellow with experience of observing paediatric clinical practice, training and expertise in interviewing, no previous experience within paediatric rheumatology, and no biases towards face-to-face, telephone, or video consultations). HS was not involved in the patients' care nor otherwise related to the patients in this study.
Occupation	3	What was their occupation at the time of the study?		
Gender	4	Was the researcher male or female?		
Experience and training	5	What experience or training did the researcher have?	Phase 1: Non-participant observations of telephone or video consultations section p5	
**Relationship with participants**				
Relationship established	6	Was a relationship established prior to study commencement?	Recruitment and Consent section pp4-5.	Participants were contacted on two occasions before data collection, as detailed in the 'Recruitment and Consent' section. Specifically, parents who expressed interest in the study shared their contact details with the research team. The researcher (HS) then initiated contact via phone or text, followed by email to provide information sheets, schedule a call, discuss the study, answer any questions, and obtain verbal consent or assent. These interactions helped

Topic	Item no.	Guide Questions/'Description	Reported in section	Commentary
				establish rapport and ensure informed participation prior to the commencement of data collection.
Participant knowledge of the interviewer	7	What did the participants know about the researcher? e.g. personal goals, reasons for doing the research	Recruitment and Consent p5	The participants were informed about the aims and content of the study. They were provided with written information and given opportunity to ask questions had to give oral and written consent to be included in this study
Interviewer characteristics	8	What characteristics were reported about the interviewer/facilitator? e.g. Bias, assumptions, reasons and interests in the research topic	Phase 1: Non-participant observations of telephone or video consultations p5	See response 1-5, especially *no previous experience within paediatric rheumatology, and no biases towards face-to-face, telephone, or video consultations*
**Domain 2: Study design**				
**Theoretical framework**				
Methodological orientation and theory	9	What methodological orientation was stated to underpin the study? e.g. grounded theory, discourse analysis, ethnography, phenomenology, content analysis	Methods section p4<br>Data analysis section p7	This is a qualitative Interpretive Description study and reflexive thematic analysis was used
**Participant selection**				
Sampling	10	How were participants selected? e.g. purposive, convenience, consecutive, snowball	Recruitment and consent section p5	A purposive sampling matrix (supplementary data, S1) targeted children aged 8 to 18 years of age, based on diagnosis, type of consultation (telephone, video) and index of deprivation.
Method of approach	11	How were participants approached? e.g. face-to-face, telephone, mail, email	Recruitment and consent section p5.	All patients were approached via telephone.<br>*Patients were screened for eligibility by a member of the rheumatology MDT (GC, SC, CP) if they had a remote consultation in the study recruitment window. Scheduled remote appointment lists were searched and if a patient met eligibility criteria the patient / family were contacted by telephone and invited to participate. A maximum of two patients*

Topic	Item no.	Guide Questions/'Description	Reported in section	Commentary
				were recruited from any particular clinic due to additional time constraints. No specific selection such as stable disease or access to digital technology was made for inclusion into the study. However, for a patient to be offered a remote consultation by the clinical team, they would in general be a follow-up patient with relatively stable disease<br>*Parents who were interested agreed to share their contact details with the research team. HS contacted parents by phone/text, and then by email to share information sheets and schedule a call to discuss the study, answer questions and obtain and document verbal consent/assent.*
Sample size	12	How many participants were in the study?	Demographics section p7 and Table 2	*Thirty-seven people participated: children and young people (n=15; male (n=7), female (n=9), age range 7-18 years, median age 12 years)*
Non-participation	13	How many people refused to participate or dropped out? Reasons?	See Demographics section p7	*No families who were contacted refused to participate, although some did not respond to initial communication. Reasons for this are not known.*
**Setting**				
Setting of data collection	14	Where was the data collected? e.g. home, clinic, workplace	Phase 1: Non-participant observations of telephone or video consultations p5<br>Demographics section, p7	*Observations were conducted via 'Attend Anywhere' video, and the researcher HS joined from home or her workplace. Observations of telephone consultations were conducted in person at the hospital.*<br>*Interviews were conducted via telephone video, according to the preferences of the participants (children, parents and health professionals). Children and parents joined calls from their homes, and health professionals from their workplace.*

Topic	Item no.	Guide Questions/'Description	Reported in section	Commentary
Presence of non-participants	15	Was anyone else present besides the participants and researchers?		Only participants and researchers were present during observations and interviews.
Description of sample	16	What are the important characteristics of the sample? e.g. demographic data, date	Demographics section p7 and Table 2	*Thirty-seven people participated: children and young people (n=15; male (n=7), female (n=9), age range 7-18 years, median age 12 years) (Table 1), parents (n=14), and HPs (n=8) (Table 2).*
**Data collection**				
Interview guide	17	Were questions, prompts, guides provided by the authors?	See Supplementary files 3, 4 and 5 and Phase 2: Interviews with HPs, parents, children and young people' p6	*Semi-structured interview guides were developed for children and young people (S3), parents (S4), and HPs (S5). Interview topics included information about themselves/their child, personal use of technology, and about the telephone or video consultation.*
		Was it pilot tested?	Involvement of experts-by-experience section p4	The interview guide was not pilot tested; however, an expert-by-experience group did provide input.
Repeat interviews	18	Were repeat interviews carried out? If yes, how many?	Phase 2: Interviews with HPs, parents, children and young people' p6	The interviews were not repeated, as the interview was about a consultation that had already occurred.<br>If a parent and child gave consent to participate in this study, an appointment for a one-time interview was made according to the preferences of the patient either a telephone or video call.<br>*Some HPs were interviewed more than once as they were part of more than one consultation with a participating child or young person.*
Audio/visual recording	19	Did the research use audio or visual recording to collect the data?	Phase 1: Non-participant observations of telephone or video consultations p5	Audio recording was undertaken.
Field notes	20	Were field notes made during and/or after the interview or focus group?	Data analysis section p7	Handwritten field notes were taken during remote consultations with children, parents and health professionals.<br>Interviews were all audio recorded with consent and transcribed verbatim by a trusted external transcription company. Handwritten observation notes were transcribed digitally, and interviews were

Topic	Item no.	Guide Questions/'Description	Reported in section	Commentary
				transcribed verbatim by a secure approved professional transcription service.
Duration	21	What was the duration of the interviews or focus group?	Demographics p7.	Interviews were designed to be in-depth but not burdensome and to last no longer than 40 minutes with adult participants (parents and health professionals) and 20 minutes with children and young people (aged 7-18years).
Data saturation	22	Was data saturation discussed?	Recruitment and consent section, p4.	We focussed not on saturation, but on the idea of data adequacy and 'information power' (34).
Transcripts returned	23	Were transcripts returned to participants for comment and/or correction?	Data analysis section, p7.	The research team purposefully decided not to return transcripts to the participants for comments and/or corrections as we wished to avoid additional research-related burden, discussed in the data analysis section
**Domain 3: analysis and findings**				
**Data analysis**				
Number of data coders	24	How many data coders coded the data?	Data analysis section, p7.	The data coding and analysis was primarily done by HS and BC.
Description of the coding tree	25	Did authors provide a description of the coding tree?	Not provided	We did not include this in the paper or supplementary data although our coding tree aligned to requirements of thematic data analysis.
Derivation of themes	26	Were themes identified in advance or derived from the data?	Data analysis section, p7.	The identified themes were derived from the data.<br><br>*Preliminary analysis of interview data was undertaken by HS using reflexive thematic analysis. Preliminary themes were discussed with the research team and with a wider group of hospital-based HPs to help ground findings in both the data and in practice. Further cycles of thematic refinement occurred (HS, BC).*

Topic	Item no.	Guide Questions/’Description	Reported in section	Commentary
Software	27	What software, if applicable, was used to manage the data?	Data analysis section, p7.	Microsoft Word was used to transcribe the interviews and work with the data. We did not use any additional software to analyse the qualitative data.
Participant checking	28	Did participants provide feedback on the findings?	Data analysis section, p7.	Participants did not provide feedback on findings as we wished to avoid additional research-related burden, especially for children. We address issues related to not using member-checking.
**Reporting**				
Quotations presented	29	Were participant quotations presented to illustrate the themes/findings? Was each quotation identified? e.g. participant number	Findings section, pp 8-11	Quotations are presented in the findings section and each quotation is appropriately identified using a clear system.<br>*D-Doctor, N-Nurse, O-Occupational Therapist, M-mother, F-father, B-boy, G-girl; digit indicates consultation (1-16) (e.g. B-8, quote from a boy in consultation 8).*
Data and findings consistent	30	Was there consistency between the data presented and the findings?	Data analysis section, p7.	We endeavoured to ensure consistency between the data presented and the findings by using quotes to support our interpretations/findings
Clarity of major themes	31	Were major themes clearly presented in the findings?	Findings section, pp 8-11<br>Figure 1<br>Table 3.	The overarching theme was that remote consultations were the ‘virtually the same but remotely different’ to face-to-face hospital-based consultations; four sub-themes were identified (see Figure 1 and Table 3).
Clarity of minor themes	32	Is there a description of diverse cases or discussion of minor themes?	Overview of findings, p8	No diverse cases were identified. We report an overarching and 4 sub-themes but did not have any further (minor) themes to report.

S2: Details about our work with experts-by-experience

Two groups of experts-by-experience provided high quality engagement and gave us confidence not to pilot the methods.

Group 1 (9 children, 4 boys, 5 girls) from GenerationR Alliance Young People's Advisory Group contributed their ideas about the study name, logo, and focus. They co-designed interviews and activity booklets in an online workshop (facilitators HS, SA, LW). Following preliminary data analysis, Group 2 (6 children, 3 boys, 3 girls, 2 mothers, 2 fathers) attended a dissemination workshop to co-design resources (animation, information leaflets) to prepare health professionals (HPs), children, young people, and parents for remote consultations.

S3: Purposive sampling matrix for REFLECT study

	Children and young people with juvenile idiopathic arthritis (N=16, age 7-18 years)			
Type of consultation	Telephone n=8		Video n=8	
Index of multiple deprivation*	Other n=4	High n=4	Other n=4	High n=4

**High indicates those in deprivation deciles 1-2 and other indicates those in deprivation deciles 3-10*

S4: Recruitment details and working definition of 'stable in disease'

Recruitment

Our planned (patient) sample size was 16 children and their parents and was estimated based on availability of suitable participants and relevant virtual clinics, the 11 month time frame for completing recruitment, and the concept of 'information power'[1]. Rather than relying on sample size as an indicator of robustness and data adequacy 'information power' considers five components (study aim, specificity of sample, use of established theory, quality of the dialogue and analysis strategy)[2]. HPs who agreed to participate were recruited and provided consent before families were approached, to avoid a situation where a child/parent consented but a HP declined.

Patients were screened for eligibility by a member of the rheumatology MDT (GC, SC, CP) if they had a remote consultation in the study recruitment window. An estimate of 80 patients were expected to be eligible for recruitment. Scheduled remote appointment lists were searched and if a patient met eligibility criteria the patient / family were contacted by telephone and invited to participate. A maximum of two patients were recruited from any individual clinic due to additional time constraints. No additional selection criteria (e.g., stable disease, access to digital technology) were considered although typically remote consultations were offered to follow-up patients with relatively stable disease.

'Stable in disease'

Note: although we did not have an a priori definition of 'stable in disease' as part of the eligibility criteria but HPs used this term when considering the suitability of children for remote ('routine') consultations. In the context of this study 'stable in disease' relates to real-world clinical judgment made by HPs based upon information gathered by history taking (telephone and video consultations) and observation of the patient (video consultations).

---

[1] Malterud K, Siersma VD, Guassora AD. Sample Size in Qualitative Interview Studies: Guided by Information Power. Qualitative Health Research. 2016;26(13):1753-60.

[2] Malterud K, Siersma VD, Guassora AD. Sample Size in Qualitative Interview Studies: Guided by Information Power. Qualitative Health Research. 2016;26(13):1753-60.

S5: Observation sheet

REFLECT

Participant ID:____________________________________ **Type of consultation:**________________________________

Date:____ **Clinic:**____

**Qualitative observations** (building the relationship, initiating the session including barriers/issues, communication, gathering information, physical examination, explanation and planning, closure)	**Questions/ things to follow up in interview**
	Where is the participant calling from?<br>What device are they using?<br>Number of devices available?<br>Did they use Wi-Fi or mobile data?<br>Images of sufficient quality?<br><br>**Date of interview:**____________________

REFLECT
EXPLORING EXPERIENCES OF VIRTUAL APPOINTMENTS

REFLECT: Exploring experiences of virtual appointments.
V1.0 07/JUL/2021 **IRAS:** 302805
Alder Hey Children's NHS Foundation Trust
Edge Hill University
University of Liverpool

S6: Semi-structured interview guides for child/young person

Introduction and check

NOTE: The process for ensuring informed assent would happen before the interview. The following is a reminder and a step put in place to gain on-going assent throughout the research process.

- Just a reminder about the interview before we start. Just now/today/yesterday/a few days ago we talked about the information leaflet and the study, and you agreed/assented to take part in the interview and for the interview to be audio-recorded.
- I'd like to check that you're still OK to do the interview. Is this still OK?
- Are you still OK if I record the things you say to me in this interview (this will help me concentrate on listening to you)?
- I might take some notes as well. Is this OK?
- OK, so that's great. Thanks so much.
- I'm interested in what you can tell me about your experience of having an appointment over the phone or by video with the [use name of person/people they saw or spoke to].
- The interview isn't a test, and there are no right or wrong answers. I'm interested in what you think. If you are unsure about a question, I can think of another way of asking it. If you want to skip a question, that's fine.
- So, have you any questions for me?
- Ok, so if you're OK with all of this, we can get started. Thanks for helping us.

Focused interview questions

NOTE: The following questions provide an indication of the questions we typically expect to ask the children/young people although we will not necessarily stick rigidly to the order or the wording indicated below.

To start off, please tell me a bit about yourself and why you had your [phone/video] appointment?

- Age/gender, disease/condition, diagnosis/duration?
- Have you had many appointments before?
- Can you remember who you saw/spoke to and what their name was?
- Have you met them before?

When you are at home, do you use a mobile phone, ipad, tablet/computer?

- Which one do you like best/why?
- Do you have to share it with other people?
- What do you use it for…?
- How often do you use it?
- Do you use it to talk to/ video call with other people? Who?
- Do you enjoy talking to other people over the phone/ by video? Why/ why not?
- Do you think it is different talking to [xxxxx] to [name of person they saw/spoke to], how
- What did you use on the call/video with [name of person/people they saw/spoke to]?
- Who was in charge/held the [xxxx]?

How do you normally see or speak to [name of person/people they saw or spoke to] or Drs/Nurses/etc? How often?

- Was your last appointment over the phone/ video?
- Was this different, why?
- Do you prefer your appointment on the phone/ by video or at the hospital? Why?

Did you know you were going to talk to/video call with [name of person they saw/spoke to] before your appointment? How did you feel about speaking with [name of person they saw/spoke to] on the phone/ video?

- OK/worried/excited/happy – why?

Can you talk to me about the person/people you saw/spoke to, can you remember who was on the call with you? How did they make you feel?

- What did [name of person they saw/spoke to] do to make you feel this way?
- Did anyone else [e.g Mum/ Dad/ guardian] do or say anything to make you feel like this?
- Do you have a top tip to share with other children about how the Dr/nurse/people could have made you feel better about being on the call/video?

Can you talk to me about your appointment, was it good/better/worse than you expected? Why/why not?

- Do you think [name of person/people they spoke to/saw and Mum/Dad] listened to you?
- Did the [name of person/people they spoke to/saw] ask you questions you think were important to you?
- Do you think you were able to talk to [name of person/people they spoke to/saw] and tell them about how you are feeling?
- Was this easier/harder over the phone/ by video? Why/why not?

Did [name of person they saw/spoke to] do any health check (examination) for your JIA with you? How did it go?

- Was this different to how your JIA was checked in your last appointment? How?
- How did the [name of person they saw/spoke to] make sure you were ready for this to happen?
- Did your Mum/Dad/Guardian stay with you for this part of your appointment? Was this okay with you?
- Can you remember what [name of person they saw/spoke to] did when they examined you?
- Did you find it easy/hard to tell/show them? Why? Is this different to when you are at the hospital with the Dr/Nurse?
- Do you think being checked in this way is better/worse than at the hospital? Why?
- Did your Mum/Dad/Guardian help you to explain/show [name of person/people they spoke to/saw], how did this make you feel?

Were there any good things about having your appointment over the phone/ by video?

Did you have any problems or issues [things that weren't so great] during your appointment?

- What were they? What happened?

- Did you have any problems connecting to the appointment? What would have helped?
- Did people help/take notice/do something? Why do you think that people missed…?
- Could you hear/ and see the [name of person/people they spoke to/saw]clearly? Could they see you? What would have helped?
- Did you have to reconnect?
- Do you normally have any problems with calls/ videos? Do you know why this is?
- Could you hear what everyone was saying? What could have helped?

Can you think of some top tips that could make the appointment over the phone/ by video better for you? What would they be?

- What would make the biggest difference to you AND to other children?
- What could adults do to make your phone/video appointment better?

Do you think appointments over the phone or by video are a good thing for children with JIA?

- Why do you think this?
- What would you tell other children with JIA about having an appointment in this way?
- What do you think children would like to know about having an appointment in this way?

Wrapping up

Checking for missing ideas

- Is there anything I have missed? Or anything else you would like to tell me about your appointment over the phone/ video?

Summing up

- What do you think is the most important thing I should remember from this interview?
- Are you OK?
- We understand that taking part in this study might make you think more about your appointment and your feelings. If you do get upset, then you might find it helpful to talk to a friend or someone you are close to. We have also created a ‘helpful information sheet’ that we would like to send to you. This sheet has the contact details of organisations that you might find helpful. Would it be ok if we sent this to you?

Thanking participant

Thanks so much for your time.

Providing a certificate for participating in the research

S7: Semi-structured interview guides for parents/carers

Introduction and check

NOTE: The process for ensuring informed consent would happen before the interview. The following is a reminder and a step put in place to gain on-going consent throughout the research process.

- Just a reminder about the interview before we start. Just now/today/yesterday/a few days ago we talked about the information leaflet and the study, and you agreed/consented to take part in the interview and for the interview to be audio-recorded.
- I'd like to check that you're still OK to do the interview. Is this still OK?
- Are you still OK if I record the things you say to me in this?
- I might take some notes as well. Is this OK?
- OK, so that's great. Thanks so much.
- I'm interested in what you can tell me about your experience of [name of child] having an appointment over the phone or by video with the [use name of person/people they saw or spoke to].
- I'm interested in what you think, if you're unsure about anything or would like me to skip a question, that's fine and if you would like to stop at anytime just let me know, that's fine.
- So, have you any questions for me?
- Ok, so if you're OK with all of this, we can get started. Thanks for helping us.

Focused interview questions

NOTE: The following questions provide an indication of the questions we typically expect to ask the parent/carer people although we will not necessarily stick rigidly to the order or the wording indicated below and will not necessarily ask every question.

To start off, please tell me a bit about [name of child] and why they had their [phone/video] appointment?

- Age/gender, disease/condition, diagnosis/duration?
- Have they had many appointments before?
- Have they had many virtual appointments before, were they mostly video calls or telephone calls?
- Can you remember who it was you saw/spoke to last time and what their name was?
- Have you met them before? Was this virtually or in person?

Overall, how do you feel that [name of child] appointment went with [name of person they saw/spoke to]?

- Why?
- Tell me more…

Does your child often use a phone, iPad, tablet/computer?

- Which one do they use most? Why?
- Do they have their own devices or do they have to share with other people? Who?
- Do they have any other devices that they share or are their own?
- What do they use their devices for…?
- Are you always with them when they use their iPad/tablet/computer?

- How often do they use it?
- Do they use it to talk to/ video call with other people? Who? Do you set the call up for them or can they/ are they allowed to do this themselves?
- Do you stay with them when they talk to/ video call with other people? Why/ why not?
- Do you think they enjoy talking to other people over the phone/ by video? Why/ why not?
- Do you think it is different talking to [xxxxx] to [name of person they saw/spoke to], how?
- What did you use on the call/video with [name of person/people they saw/spoke to]?
- Who was in charge/held the [xxxx]? How did you decide this?

Do you often use a phone, iPad, tablet/computer?

- Which one do you use most? Why?
- What do you use the device for? E.g work/banking/video calls with family
- How confident are you using a phone/iPad, tablet/computer? Why? Why not?
- What device did you use for your child's appointment? Why did you choose to use this device? Were other options available?

Did you know [name of child] were going to talk to/video call with [name of person they saw/spoke to], before your appointment? How did you feel when you realised that your appointment wasn't going to be face to face at the hospital but by phone/video? Why?

- OK/worried/excited/happy/shy – why?
- Would you have preferred the appointment to have been at the hospital, in person? Or by video (if by phone) or by phone (if by video), why?

How does [name of child] normally see or speak to [name of person/people they saw or spoke to] or Drs/Nurses/etc? How often?

- Was your last appointment over the phone/ video?
- Was this similar/different, why?
- Do you think there are any benefits/drawbacks of your child having an appointment by [phone/video]?
- Do you prefer your appointment on the phone/ by video or at the hospital? Why?

Can you talk to me about being on the [phone/video] with [the person/people you spoke to/saw], can you talk me through what happened?

- Does anything stand out as good or not so good about your [name of child] having a [phone/video] appointment?
- How did you feel during the [phone/video] appointment?
- Did [name of person they saw/spoke to] do anything in particular to make you feel this way?
- Do you think you were listened to?
- Do you think [name of child] felt listened to? Why/ why not?
- Did you say or do anything to [name of child] during the call to help them? What did you do/say? Can you remember what happened after this?
- Do you have any comments about how the Dr/nurse/people could have made the [phone/video] appointment better for you and for your child?

Did you have any problems or issues [things that weren't so great] during your appointment?

- What were they? What happened?
- Did you have to use wi-fi or your mobile data to connect to the call, at all? Did you have any problems connecting to the appointment? What would have helped?
- Did people help/take notice/do something? Why do you think that people missed…?
- Could you hear/ and see the [name of person/people they spoke to/saw] clearly? Could they see you? What was the quality of the connection like? What would have helped?
- Did you have to reconnect?
- Do you normally have any problems with calls/ videos? Do you know why this is?
- Could you hear what everyone was saying clearly? What could have helped?

Was it good/better/worse than you expected? Why/why not?

- Did the [name of person/people they spoke to/saw] ask you questions you think were important to you? Can you remember what they were?
- Did [name of person/people they spoke to/saw] ask your child questions you think were important to them? Can you remember what they were?
- Did you feel you were able to talk to [name of person/people they spoke to/saw] and tell them about any concerns or ask any questions you might have about your child's health? Why/why not?
- Was this easier/harder over the phone/ by video? Why?
- Were there any good things about having your appointment over the phone/by video?

Did [name of person they saw/spoke to] examine [name of child]? How did it go?

- Was this different to how they were examined in their last appointment? How?
- How did the [name of person they saw/spoke to] make sure [name of child] was ready for the examination?
- How do you think the examination went? Was this better/worse than previous appointments (face to face or virtually)? How?
- Did you stay with [name of child] for this part of your appointment? Why/why not?
- Do you normally stay for their examinations or is this different to at the hospital?
- Can you tell me what [name of person they saw/spoke to] did to examine [name of child]?
- Did [your child] find it easy/hard to tell/show them? Why? Is there anything that could have helped?
- Do you think being examined in this way is better/worse than at the hospital? Why?
- Did you help [name of child] explain/show [name of person/people they spoke to/saw], what did you do? How did this help?
- Where were you and [name of child] for the [call/video]?
- Do you think your child had enough privacy during the examination? Why/ why not?

Do you think appointments over the phone or by video are a good thing for children with JIA and their parents?

- Why do you think this?
- What would you tell other parents with children with JIA about having an appointment in this way?

In relation to the Drs/Nurses/people doing your child's appointment, is there anything you think we could improve in the future?

- What would make the biggest difference to you, your child and to other children?
- What could make having a phone/video appointment better?
- Would you be okay having more appointments this way in the future? Why/ Why not?

Wrapping up

Checking for missing ideas

- Is there anything I have missed? Or anything else you would like to tell me about your child having an appointment over the phone/ video?

Summing up

- What do you think is the most important thing I should remember from this interview?
- Are you OK?
- We understand that taking part in this study might make you think more about your appointment and your feelings. If you do get upset, then you might find it helpful to talk to a friend or someone you are close to. We have also created a 'helpful information sheet' that we would like to send to you. This sheet has the contact details of organisations that you might find helpful. Would it be ok if we sent this to you?
- Have you got any more questions?

Thanking participant

- Thanks so much for your time.

S8: Semi-structured interview guides for Health Professionals

Introduction and check

NOTE: The process for ensuring informed consent would happen before the interview. The following is a reminder and a step put in place to gain on-going consent throughout the research process.

- Just a reminder about the interview before we start. Just now/today/yesterday/a few days ago we talked about the information leaflet and the study, and you agreed/consented to take part in the interview and for the interview to be audio-recorded.
- I'd like to check that you're still OK to do the interview. Is this still OK?
- Are you still OK if I record the things you say to me in this?
- I might take some notes as well. Is this OK?
- OK, so that's great. Thanks so much.
- I'm interested in what you can tell me about your experience of conducting an appointment over the phone or by video with the [use name of person/people they saw or spoke to].
- I'm interested in what you think, if you're unsure about anything or would like me to skip a question, that's fine and if you would like to stop at anytime just let me know, that's fine.
- So, have you any questions for me?
- Ok, so if you're OK with all of this, we can get started. Thanks for helping us.

Focused interview questions

NOTE: The following questions provide an indication of the questions we typically expect to ask the clinician although we will not necessarily stick rigidly to the order or the wording indicated below and not all questions will necessarily be asked.

To start off, please tell me a bit about you, your job and role in the hospital?

- (Position, duration in post/experience)
- Do you use a phone, iPad/computer much at home too? Which ones? Why/why not? What for?
- Do you enjoy using phone or video for keeping in touch with friends and family. Why?

How often would you say you conduct phone/video appointments?

- Has this changed as lockdown has been eased more and more? Is this because you wanted to make the change or because it was required? Please tell me more…
- Did you conduct appointments this way before the pandemic? How often?
- Do you tend to conduct phone or video appointments? Why is this? What do you like/ don't you like about the technology?
- What do you use to conduct the appointment? (e.g personal computer, shared computer, iPad etc). Why?
- Do you think it is different talking to your patients and their parents in this way? How?
- How do you feel when you know you have a morning or afternoon when your whole clinic is by phone/video? How is this different to the way you feel approaching a clinic, face to face at the hospital?

Thinking back to your appointment with [name of child], how well do you know the [name] and their parents?

- Has [name of child] had many appointments with you before?
- Have these appointments been face-to-face/phone/video?
- Was your last appointment with [name of child] over the phone/ video?
- Was this different, why?
- Do you think there are any benefits/drawbacks having an appointment by [phone/video] specifically with [name of child]?
- Would you prefer your appointment with [name of child] to be conducted on the phone/ by video or at the hospital? Why?
- Do you feel under pressure from other people/systems to conduct appointments using a particular mode? Please tell me more.

Can you talk to me about being on the [phone/video] with [name of child], can you talk me through what happened?

- How do you feel the appointment went? Why?
- Does anything stand out as good or not so good about the appointment with [name of child]?
- Do you think [name of child] and [name of parent/role of parent] felt listened to? Why/ why not?
- Do you think there is anything that YOU could have done to make the appointment better for [name of child] or their parent/carer?
- Do you think there is anything that the hospital/other people [not child/parents] could have done to make the appointment better for [name of child] or their parent/carer?
- Is there anything you think could have made the appointment better for you?

Did you have any problems or issues during the appointment?

- What were they? What happened? Are these frequent/regular/expected/unusual? Do you normally have any problems with calls/ videos? Do you know why this is?
- Did you have any problems connecting to the appointment? What would have helped?
- Did [name of child/parent] have any problems connecting to the appointment? Did you do anything to help?
- Do you think [name of child] and their parent/carer understood how to connect to the call/video and use the device? Did they seem /say they were stressed at the start of the appointment as a result of connection/other problems?
- Could you hear/ and see the [name of child] clearly? Could they see you? What would have helped?

Were you able to examine [name of child]? How did it go?

- Was this different to how they were examined in their last appointment? Was this last appointment remote? How was it different?
- How did you make sure [name of child] was ready for the examination?
- How do you think the examination went? Was this better/worse than previous appointments? How?
- Did [name of child] parent/carer stay with them for this part of your appointment? Were you okay with this?
- Do you think [name of child] was okay with this? Why/why not?

- Do you think [name of child] had enough privacy during their examination? Why/ why not? What could have helped?
- Can you tell me what you did to examine [name of child]? How did you instruct [name of child] what to tell you/show you?
- Did [name of child] find it easy/hard to tell/show you? Why? Is there anything that could have helped?
- Do you think being examined in this way is better/worse/ more difficult than at the hospital? Why?
- Did the [name of child] parent/carer help [name of child] to explain/show you, what did they do? Do you think this as helpful? Why/ why not?

Overall, what is your opinion of conducting phone/video appointments, in general?

- Why do you feel this way?
- Has this changed?
- What works well/ not so well about them? Is there anything that is particularly frustrating?
- Are you able to ask the questions and get answers from children that you need?
- Is there anything you find difficult about conducting phone/video appointments? Is there anything that could help? E.g training, better technology, better….
- Do you think children and parents feel able to talk to you and ask you questions over the phone/video? Do you think this is easier/ harder over the phone/by video? Why?
- What do you think others (e.g HCPs, children, parents) think of conducting phone/video appointments?
- How would you make a decision to decide if a child should attend Alder Hey for an appointment or be scheduled for either phone or video?
- Do you think that there is a hierarchy of 'best' appointments (could you rank what's best out of hospital, video, telephone) Why?

Are there any good things about conducting appointments over the phone/ by video?

Do you think appointments over the phone or by video are a good thing for children with JIA

- Why do you think this?
- If you had to look in to the future of appointments for children what do you see? How do you think they will be conducted? What do you think the benefits are of this? Do you think there are any challenges with this?

Wrapping up

Checking for missing ideas

- Is there anything I have missed? Or anything else you would like to tell me about conducting an appointment over the phone/ video?

Summing up

- What do you think is the most important thing I should remember from this interview?
- Are you OK?
- Have you got any more questions?

Thanking participant

- Thanks so much for your time.

Email (if consented to providing this information) a certificate for portfolio

S9: Activity booklets used pre-interview to prepare children and young people

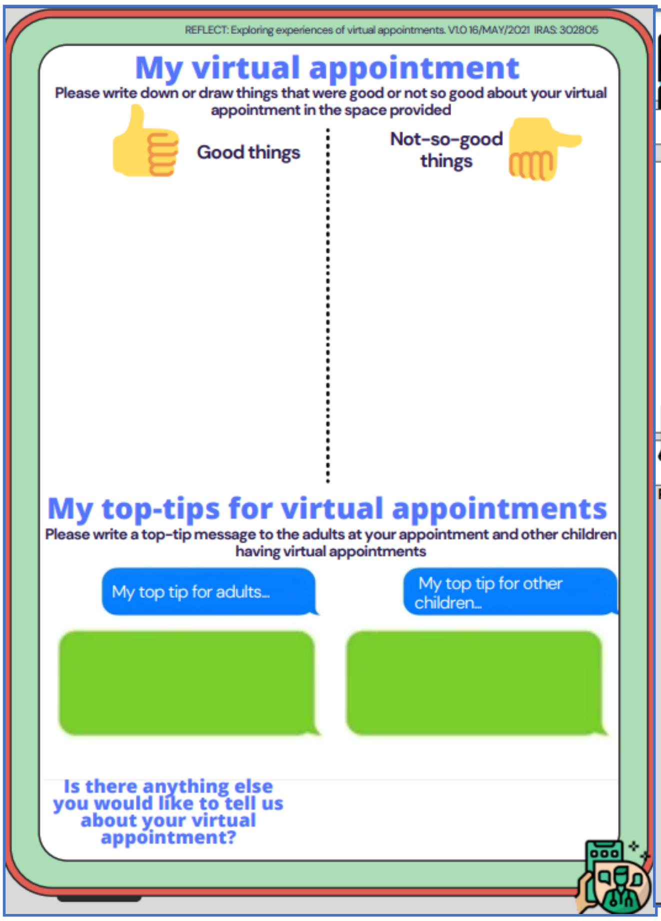

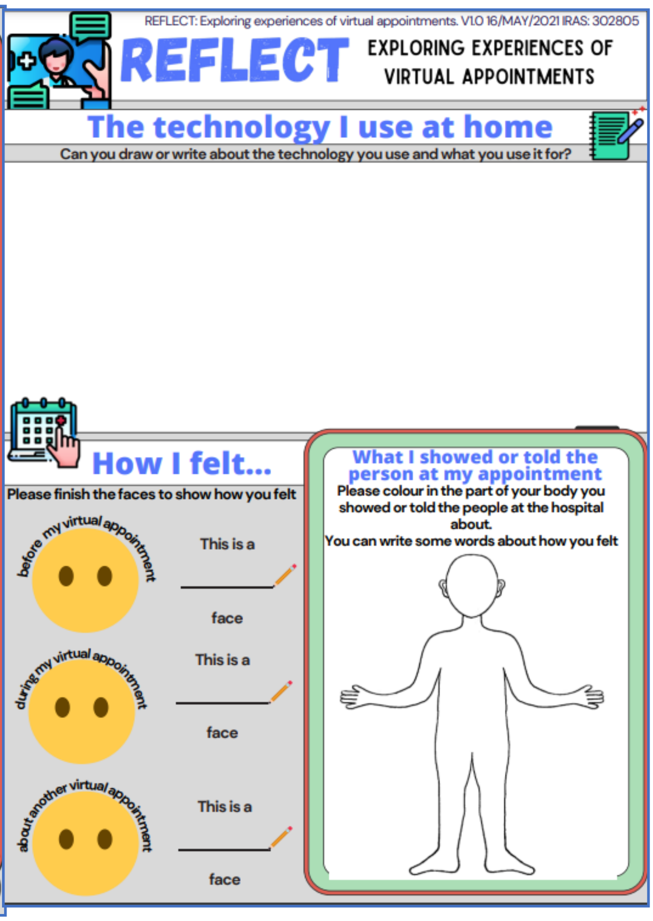

S10: Ethics and governance

Ethics approval was gained via HRA and Health and Care Research Wales (HCRW) (IRAS ID 302805) as well as via the Health Research Ethics Committee, Edge Hill University.

The security for the telephone and video consultations complied with stringent security measures as governed by Alder Hey Children's NHS Foundation Trust Information Governance Policy. The interviews were undertaken via secure, password-protected platforms. Confidentiality and anonymity of all participants was ensured through clear standard operating procedures, including complying with the UK General Data Protection Regulation[3] which requires adherence to seven key principles for handling personal data, including data minimisation. Recordings of observations and interviews were audio-only.

All participants were made aware of how we planned to protect anonymity via the specific Participant Information Sheets. All personally identifying details (e.g. personal information and unique characteristics) were removed from transcripts and survey data to avoid 'deductive disclosure'[2], in line with best practices. Direct quotations are not traceable to the individual participants. Study specific numeric identifiers were used to protect participant identities. Participants were aware of the limits to confidentiality, as we noted that safeguarding processes would be followed if concern arose about a participant's safety, the safety of someone else, or if poor practice was witnessed[4].

All data were stored in encrypted storage drives/designated storage sites within the settings with only anonymised data allowed to flow between the hospital and university sites.

---

[3] ICO. UK GDPR guidance and resources: Information Commissioner's Office; 2024 [Available from: https://ico.org.uk/for-organisations/uk-gdpr-guidance-and-resources/.

[4] UK Statistics Authority. Ethical considerations associated with Qualitative Research methods: confidentiality and data security: UK Statistics Authority; 2022 [Available from: https://uksa.statisticsauthority.gov.uk/publication/ethical-considerations-associated-with-qualitative-research-methods/pages/6/.

S11: Process for ensuring quality of data

Although we did not undertake member-checking as we wished to avoid additional research-related burden[5], particularly for the child participants, we took measures to ensure the quality of the transcripts. During the interview HS checked her understanding of specific responses and gained clarification as required and provided participants with the opportunity to clarify any issues or add any further information and context. Transcriptions were checked within two weeks of the interview. These processes along with field notes helped ensure accuracy and credibility of the data.

[5] Luchtenberg ML, Maeckelberghe ELM, Locock L, Verhagen AAE. Understanding the child-doctor relationship in research participation: a qualitative study. BMC Pediatrics. 2020;20(1):353.

S12: Reflexivity

Reflexivity is core to transparent research practice as it helps to account for how subjectivity has shaped the research[6,7]. Both individually and within team meetings we deliberately considered and challenged assumptions, beliefs and positions throughout the study. Our core team, two consultants (one male, one female) in paediatric rheumatology, an academic children's nurse (female), and a social scientist (female, who undertook the observation and interviews) brought diversity of knowledge, skills and expertise; this encouraged active questioning and insights as we learned, shared and reflexively questioned. Analysis was led by the academic children's nurse (female) and the social scientist and supported by the consultants before being shared with the wider team. Our wider team consisted of professionals and academics from a wide range of disciplinary backgrounds and acted as a 'sounding box' for fruitful and critical conversations about methodology, methods, analysis and context. These reflexive conversations were particularly dynamic as we refined the themes, identified core concepts for the discussion and conclusion. These conversations and a commitment to reflexivity also informed the development of the resources we developed as part of the study.

[6] Eck K, Lanigan A. Doing Reflexivity. Uppsala universitet, Institutionen för freds- och konfliktforskning Aalborg University APSA Organized Section on Qualitative Methods/Consortium for Qualitative Research Methods; 2024

[7] Olmos-Vega FM, Stalmeijer RE, Varpio L, Kahlke R. A practical guide to reflexivity in qualitative research: AMEE Guide No. 149. Medical Teacher. 2023;45(3):241-51.
